# Early Myocardial Deformation Changes in Hypercholesterolemic and Obese Children and Adolescents: A 2D and 3D Speckle Tracking Echocardiography Study

**DOI:** 10.1097/MD.0000000000000071

**Published:** 2014-09-05

**Authors:** Antonio Vitarelli, Francesco Martino, Lidia Capotosto, Eliana Martino, Chiara Colantoni, Rasul Ashurov, Serafino Ricci, Ysabel Conde, Fabio Maramao, Massimo Vitarelli, Stefania De Chiara, Cristina Zanoni

**Affiliations:** Department of Cardiology (AV, LC, RA, YC, FaM); Department of Pediatrics (FrM, EM, CC, CZ); and Department of Medicine (SR, MV, SDC), Sapienza University, Rome, Italy.

## Abstract

Dyslipidemia and obesity are considered strong risk factors for premature atherosclerotic cardiovascular disease and increased morbidity and mortality and may have a negative impact on myocardial function.

Our purpose was to assess the presence of early myocardial deformation abnormalities in dyslipidemic children free from other cardiovascular risk factors, using 2-dimensional speckle tracking echocardiography (2DSTE) and 3-dimensional speckle tracking echocardiography (3DSTE).

We studied 80 consecutive nonselected patients (6–18 years of age) with hypercholesterolemia (low-density lipoprotein [LDL] cholesterol levels >95th percentile for age and sex). Forty of them had normal weight and 40 were obese (body mass index >95th percentile for age and sex). Forty healthy age-matched children were selected as controls. Left ventricular (LV) global longitudinal, circumferential, and radial strains were calculated by 2DSTE and 3DSTE. Global area strain (GAS) was calculated by 3DSTE as percentage of variation in surface area defined by the longitudinal and circumferential strain vectors. Right ventricular (RV) global and free-wall longitudinal strain and LV and RV diastolic strain rate parameters were obtained. Data analysis was performed offline.

LV global longitudinal strain and GAS were lower in normal-weight and obese dyslipidemic children compared with normal controls and reduced in obese patients compared with normal-weight dyslipidemic children. LV early diastolic strain rate was lower compared with normals. RV global and free-wall longitudinal strain was significantly reduced in obese patients when compared with the control group. A significant inverse correlation was found between LV strain, LDL cholesterol levels, and body mass index.

2DSTE and 3DSTE show LV longitudinal strain and GAS changes in dyslipidemic children and adolescents free from other cardiovascular risk factors or structural cardiac abnormalities. Obesity causes an additive adverse effect on LV strain parameters and RV strain impairment.

## INTRODUCTION

Dyslipidemia and obesity are considered strong risk factors for premature atherosclerotic cardiovascular disease and increased morbidity and mortality and may have an adverse effect on left ventricular (LV) performance.^1–5^ Two-dimensional speckle tracking echocardiography (2DSTE) allows the assessment of subclinical cardiac dysfunction in different diseases on the basis of myocardial deformation parameters.^6,7^ Reductions in longitudinal and circumferential deformation were demonstrated in children with heterozygous familial hypercholesterolemia,^8^ and left and right systolic–diastolic ventricular impairment using 2-dimensional (2D) speckle tracking longitudinal strain has also been described in obese children and adolescents without comorbidities.^9^ Three-dimensional speckle tracking echocardiography (3DSTE) provides additive information regarding different parameters of LV myocardial deformation.^10–12^ Our aim was to assess the presence of early myocardial abnormalities using 2DSTE and 3DSTE in nonselected normal-weight and obese dyslipidemic children and adolescents free from other cardiovascular risk factors.

## METHODS

### Population

Eighty consecutive nonselected patients (6–18 years of age, 45 men) with hypercholesterolemia (low-density lipoprotein [LDL] cholesterol levels >95th percentile for age and sex) were enrolled. Forty of them had normal weight and 40 were obese (body mass index >95th percentile for age and sex). Mean age was 10.48 ± 3.42 and 10.74 ± 3.67 years in the normal-weight and obese groups, respectively. None of them had any other cardiovascular risk factors. Children with thyroid dysfunction, nephrotic syndrome, autoimmune disease, liver disease, primary biliary cirrhosis, and sleep apnea (according to parents’ information) were excluded. Forty healthy children matched for age and sex were also recruited. Systolic and diastolic blood pressures were systematically measured during the echocardiographic studies. The study was approved by the local ethics committee, and written informed consent was obtained from all subjects.

### Two-Dimensional Echocardiography

Patients were examined in the left lateral decubitus position using a Vivid E9 commercial ultrasound scanner (GE Vingmed Ultrasound AS, Horten, Norway) with an active matrix single-crystal phased-array transducer (GE M5S-D; GE Vingmed Ultrasound AS). Grayscale recordings were optimized at a mean frame rate of ≥50 frames/s. Measurements of cardiac chambers were made by transthoracic echocardiography according to established criteria.^13^ Peak early (E) and late (A) diastolic velocities, deceleration time, LV isovolumic relaxation time, myocardial performance index, and right ventricular (RV) systolic pressure were obtained using standard Doppler practices. Mitral annular velocities (S_a_, E_a_, and A_a_) were measured on the transthoracic 4-chamber views.

**FIGURE 1:**
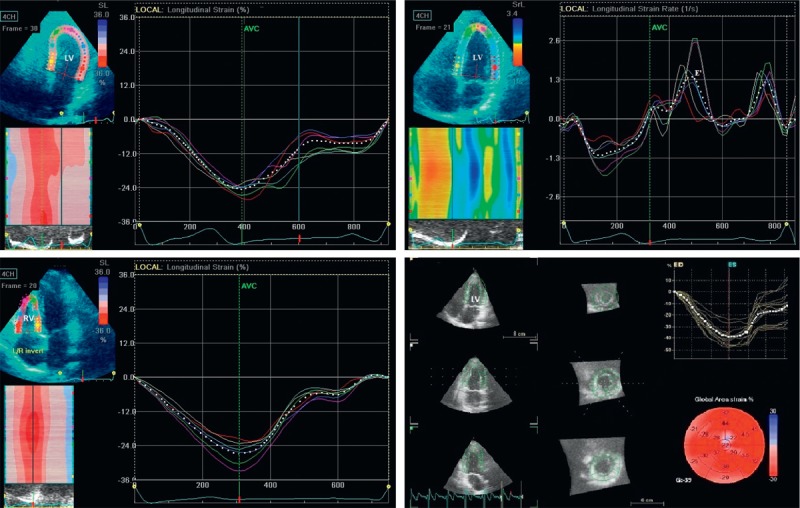
Representative 2D and 3D strain images in normal controls. 2D LV longitudinal strain (−24%, top left). 2D LV longitudinal strain rate (top right). E′_STE_ (1.32 s^−1^). 2D RV longitudinal strain (−27%, bottom left). 3D LV GAS (−39%, bottom right). 2D = 2-dimensional, 3D = 3-dimensional, E′_STE_ = peak early diastolic longitudinal strain rate, GAS = global area strain, LV = left ventricular, RV = right ventricular.

LV 2D longitudinal strain (Figure 1) was calculated in 3 apical views in relation to the strain value at aortic valve closure and measured in 17 segments on the basis of the software Bullseye Diagram. Strain values were not derived in the presence of >2 suboptimal segments in a single apical view. Longitudinal systolic deformation was characterized as shortening, and systolic indices provided negative values. Circumferential and radial systolic strains were calculated as an average of strain values obtained from the basal, mid, and apical parasternal short-axis views. The global LV longitudinal peak early diastolic strain rates were measured in the apical 4-chamber view (Figure 1) by regarding the entire length of the visualized LV wall, and the peak early diastolic longitudinal strain rate (E′_STE_) was used for calculating the E/E′_STE_. Manual readjustments were made only when necessary to ensure accurate tracking.

To assess regional and global RV systolic function in the longitudinal direction, we adopted a 6-segment RV model (basal RV lateral wall, mid RV lateral wall, apical RV wall, apical septum, mid septum, and basal septum). Peak systolic strain was recorded for the 3 RV myocardial free-wall and septal segments and the entire RV wall (Figure 1). The following measurements were obtained: free-wall right ventricular longitudinal strain (FW-RVLS), global right ventricular longitudinal strain (RVLS), and RV longitudinal early diastolic strain rate (RV-E′_STE_). Global strain and strain rate were calculated by averaging local strains along the entire right ventricle, using software (EchoPAC BT12; GE Vingmed Ultrasound AS).

### Three-Dimensional Echocardiography

A fully sampled matrix-array transducer with almost 3000 active elements was used (4V-D; GE Vingmed Ultrasound AS). The acquisition of 3-dimensional echocardiography (3DE) data was obtained in an adjustable volume divided into 6 subvolumes. By keeping the ultrasonic transducer in a stable position, the acquisition of subvolumes was steered electronically and triggered to the ECG R wave on consecutive heartbeats. Acquisitions were recorded at the LV apex during end-expiration breath-hold with a mean volume rate of ≥30 vol/s and a 6-beat acquisition to obtain a correct spatial registration of all subvolumes and optimal temporal–spatial resolution. To optimize the acquisition frame rate ≥30 Hz (30 frames/s), depth was minimized to include only the left ventricle.

Offline data analysis was determined on a separate workstation for the software (EchoPAC BT12, 4D Auto LVQ; GE Vingmed Ultrasound AS), using the original raw data from 3-dimensional data sets. Alignment was performed with the presentation of 4-chamber, 2-chamber, and 3-chamber apical views, as well as short-axis views. For the end-diastolic volumes, the operator placed one point in the middle of the mitral annular plane and a second point at the LV apex, generating an end-diastolic endocardial border tracing and including the papillary muscles within the LV cavity. For the end-systolic volumes, the same process was repeated in end-systole, and acquisition of LV volumes and left ventricular ejection fraction (LVEF) was obtained. The correct alignment of the endocardial contours during the cardiac cycle was checked to obtain the volume waveform. A second semiautomated epicardial tracking was made to delineate the region of interest for strain analysis (3DSTE)*.* 3DSTE was used to determine at end-systole global longitudinal strain (GLS), global circumferential strain (GCS), global area strain (GAS), and global radial strain (GRS). GAS was determined as the percentage of decrease in the size of endocardial surface area defined by the vectors of longitudinal and circumferential deformations. Following a frame-by-frame analysis, a final 17-segment Bullseye map of strain values was displayed (Figure 1). Global strain values were automatically calculated by the software and were not determined in the presence of >3 uninterpretable segments.

### Statistics

Data are presented as mean value ± SD. Linear correlations and univariate and multivariate analyses were used for comparisons. Multivariate analyses were performed using a stepwise forward regression model in which each variable with *P* < 0.1 or less on univariate analysis was entered into the model. Variance inflation factor approach was used to identify collinearity among explanatory variables. Variables were compared among groups by Student *t* test. Differences were considered statistically significant if the *P* value was <0.05. Intraobserver and interobserver variabilities of strain measurements were evaluated in 10 randomly selected patients. To analyze intraobserver variability, measurements of strain parameters were made at multiple sites in different patients on 2 different occasions. For interobserver variability, a second investigator randomly made measurements at the above different sites without knowledge of other echocardiographic parameters. The intraobserver and interobserver variabilities were determined as the difference between the 2 sets of observations divided by the mean of the observations and expressed as a percentage. Beat-to-beat variability was assessed by the analysis of multiple 2D and 3D loops in a subset of 10 randomly selected normal subjects. Acquisition variability was assessed by repeating the test with a different operator within 1 hour after the first study without alteration of hemodynamics or therapy and analyzing by separate observers the measurements of the same strain parameters obtained in the 2 different acquisitions.

## RESULTS

Eighty out of 91 initially evaluated patients were included in the study. Eleven patients were excluded due to inadequate myocardial tracking (n = 7 both 2DSTE and 3DSTE, n = 3 only 3DSTE) or rhythm abnormalities (n = 1). Global feasibility of the study was 88%. The intraobserver and interobserver variabilities were slightly higher for 2D GLS, GCS, and GRS compared with the corresponding 3D strain values. For 2DSTE, intraobserver variability was 6.3% ± 3.1% for FW-RVLS, 7.4% ± 3.5% for GLS, 8.9% ± 3.4% for GCS, 11.5% ± 4.6% for GRS, 7.1% ± 3.2% for E′_STE_, and 6.7% ± 3.2% for RVLS, and interobserver variability was 6.9% ± 3.2% for FW-RVLS, 8.6% ± 3.9% for GLS, 9.7% ± 3.7% for GCS, 12.9% ± 4.5% for GRS, 7.9% ± 3.6% for E′_STE_, and 7.3% ± 3.4% for RVLS. For 3DSTE, intraobserver variability was 6.6% ± 2.3% for GLS, 7.8% ± 2.7% for GCS, 5.7% ± 2.4% for GAS, and 9.7% ± 4.1% for GRS, and interobserver variability was 7.3% ± 2.8% for GLS, 8.4% ± 2.9% for GCS, 6.1% ± 2.6% for GAS, and 10.2% ± 3.9% for GRS. Beat-to-beat variability on average had a coefficient of variance <10%. Acquisition variability for 2DSTE was 8.2% ± 4.1% for GLS, 9.8% ± 3.9% for GCS, 11.8% ± 5.2% for GRS, 8.1% ± 3.3% for E′_STE_, 6.8% ± 3.5% for FW-RVLS, and 6.9% ± 3.7% for RVLS, and for 3DSTE was 7.1% ± 2.2% for GLS, 8.5% ± 2.9% for GCS, 6.3% ± 2.4% for GAS, and 10.1% ± 4.1% for GRS.

The baseline characteristics of patients and controls are listed in Table 1. There were no significant differences between groups with regard to age and sex. Blood pressure values in obese patients were slightly higher, showing a statistical trend toward significance (*P* = 0.078).

**TABLE 1:**
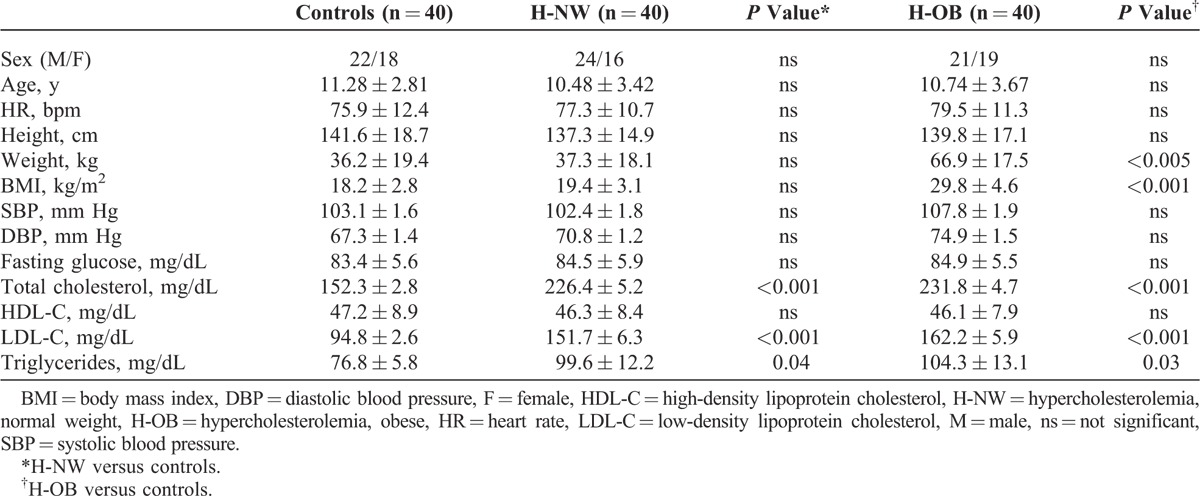
Clinical Characteristics

Dyslipidemic children had increased LV-wall thickness and mass index compared with controls (Table 2). LVEF was similar for all groups. Prolonged mitral inflow deceleration time, reduced early diastolic mitral annular velocity, and higher early transmitral/early diastolic mitral annular velocity ratios were shown in patient groups.

**TABLE 2:**
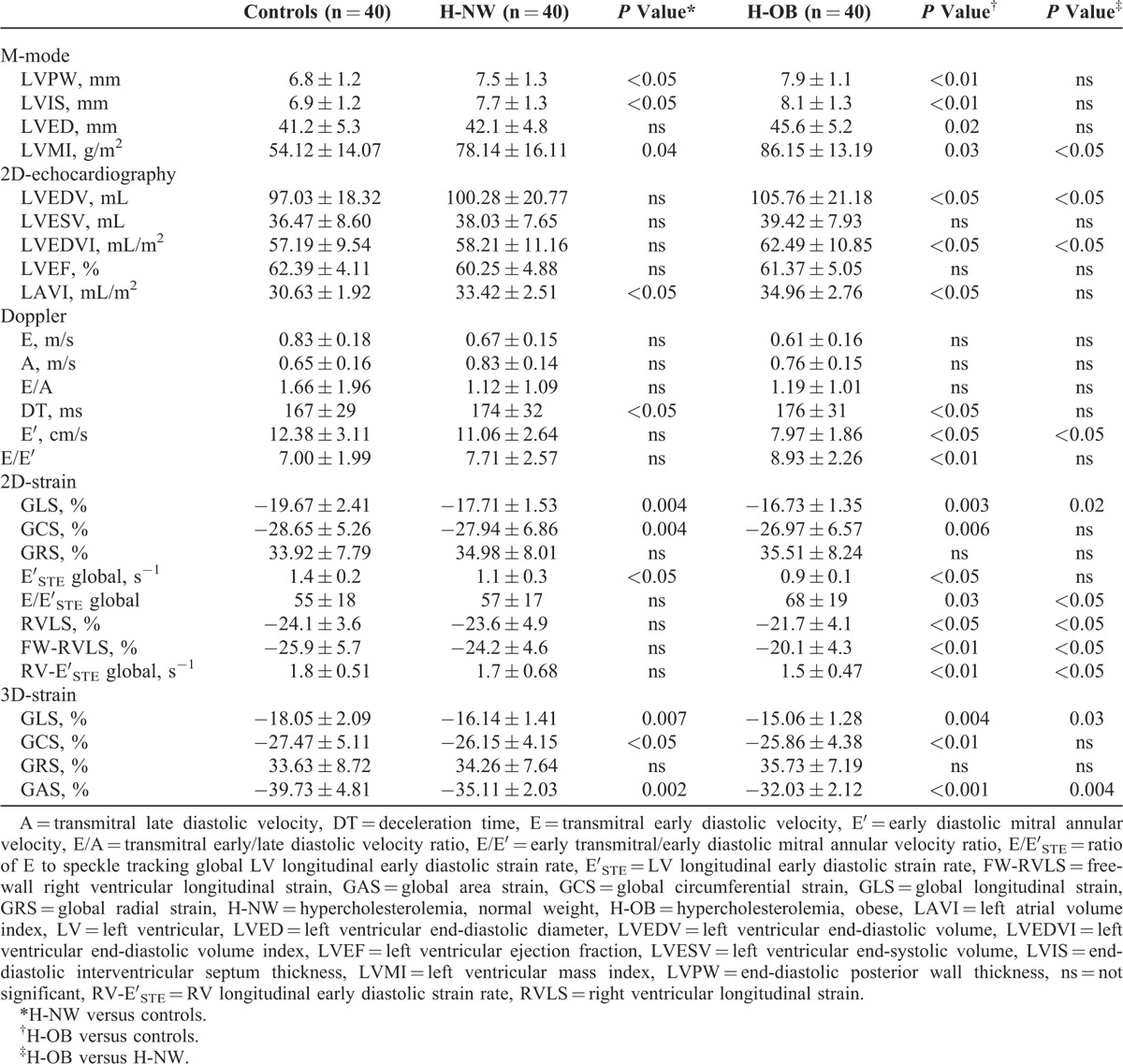
Echocardiographic Characteristics

**FIGURE 2:**
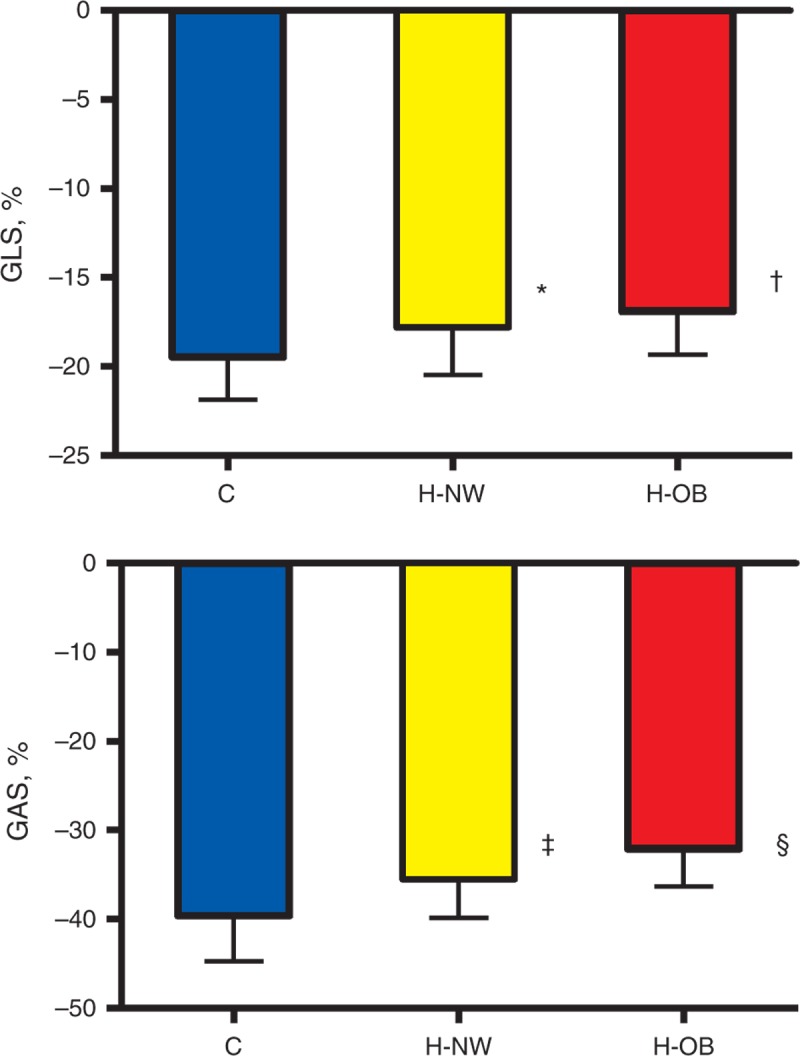
Comparison of mean GLS 2DSTE-derived (top) and area strain (bottom) in normal controls, H-NW, and H-OB. *P < 0.005 H-NW versus controls, †*P* < 0.01 H-OB versus H-NW, ‡*P* < 0.0001 H-NW versus controls, and §*P* < 0.005 H-OB versus H-NW. C = normal controls, GAS = global area strain, GLS = global longitudinal strain, H-NW = normal-weight hypercholesterolemic children, H-OB = obese hypercholesterolemic children.

LV myocardial 2D and 3D deformation parameters were significantly reduced in normal-weight and obese dyslipidemic children compared with normal controls and in obese patients compared with normal-weight dyslipidemic children (Table 2, Figure 2). E′_STE_ was lower compared with normals both in normal-weight and obese dyslipidemic patients. E/E′_STE_ was higher only in the obese group (Table 2). No correlation was found between LV mass index and strain parameters (data not shown). RV deformation parameters (RV global and free-wall indices and RV early diastolic strain rate) were significantly reduced in obese patients compared with the control group (Table 2).

The total duration of 3D data analysis averaged 9.1 ± 1.5 minutes, which was 49% less than the time used for 2D analyses (14.7 ± 2.4 minutes, *P* < 0.005). Both image acquisition time (2.8 ± 0.7  vs 4.6 ± 1.1 minutes, *P* < 0.005) and offline analysis time (6.3 ± 0.8  vs 10.1 ± 2.3 minutes, *P* < 0.001) were significantly faster for 3DSTE compared with 2DSTE. Analysis time included calculation of LV volumes, LVEF, and all 3 (2D) or 4 (3D) strains from a single vendor-specific algorithm.

Global LS and CS measured by 2DSTE and 3DSTE showed significant correlations between both methods (LS, *r* = 0.88, *P* < 0.001; CS, *r* = 0.84, *P* < 0.005). No significant correlation between global RS extracted from 3DSTE and 2DSTE was found. The 3DSE approach gave lower values than 2DSTE for both global LS and CS components (Table 2). The correlation coefficient for segmental strain measured with 2DSTE and 3DSTE was 0.62 (*P* < 0.01) for LS and 0.44 (*P* < 0.05) for CS. When comparing segmental LS measured with 2D and 3D methods at ventricular levels, the smallest differences were found in the midventricular segments (*P* = ns), whereas significantly larger differences were obtained in the basal (*P* < 0.01) and apical (*P* < 0.05) segments.

**FIGURE 3:**
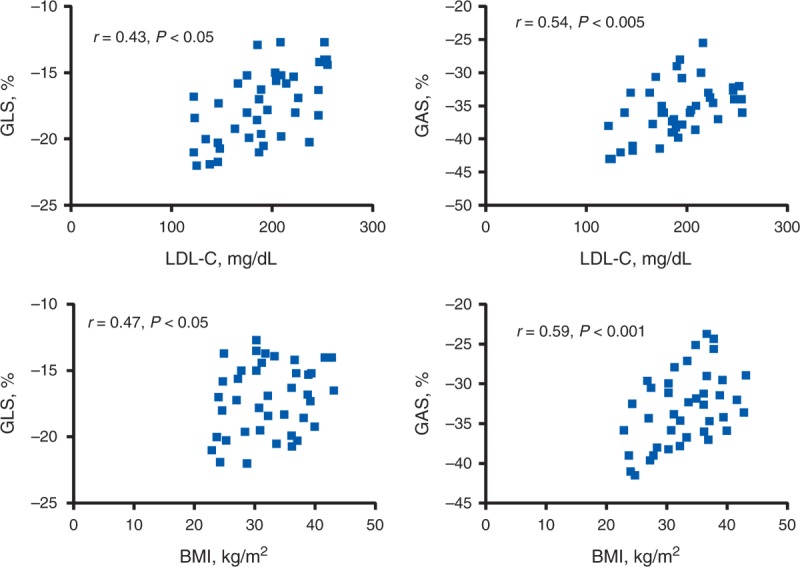
H-NW (top): linear correlation between 2DSTE-derived GLS and LDL cholesterol level (top, left) and 3DSTE-derived GAS and LDL-C level (top, right). H-OB (bottom): linear correlation between 2DSTE-derived GLS and BMI level (bottom, left) and 3DSTE-derived GAS and BMI (bottom, right). 2DSTE = 2-dimensional speckle tracking echocardiography, 3DSTE = 3-dimensional speckle tracking echocardiography, BMI = body mass index, GAS = global area strain; GLS = global longitudinal strain, H-NW = normal-weight hypercholesterolemic children, H-OB = obese hypercholesterolemic children, LDL-C = low-density lipoprotein cholesterol.

Significant inverse correlations (Figure 3) were found between LDL cholesterol levels and 2D-derived longitudinal strain (*r* = 0.43, *P* < 0.05), LDL cholesterol levels and 3D-derived longitudinal strain (*r* = 0.41, *P* < 0.05), LDL cholesterol levels and GAS (*r* = 0.54, *P* < 0.005), body mass index and 2D-derived longitudinal strain (*r* = 0.47, *P* < 0.05), body mass index and 3D-derived longitudinal strain (*r* = 0.44, *P* < 0.05), and body mass index and GAS (*r* = 0.59, *P* < 0.001). By multivariate analysis, 2D-GLS (*P* = 0.038), 3D-GLS (*P* = 0.044) and GAS (*P* = 0.012) were independently associated with hypercholesterolemia (Table 3).

**TABLE 3:**
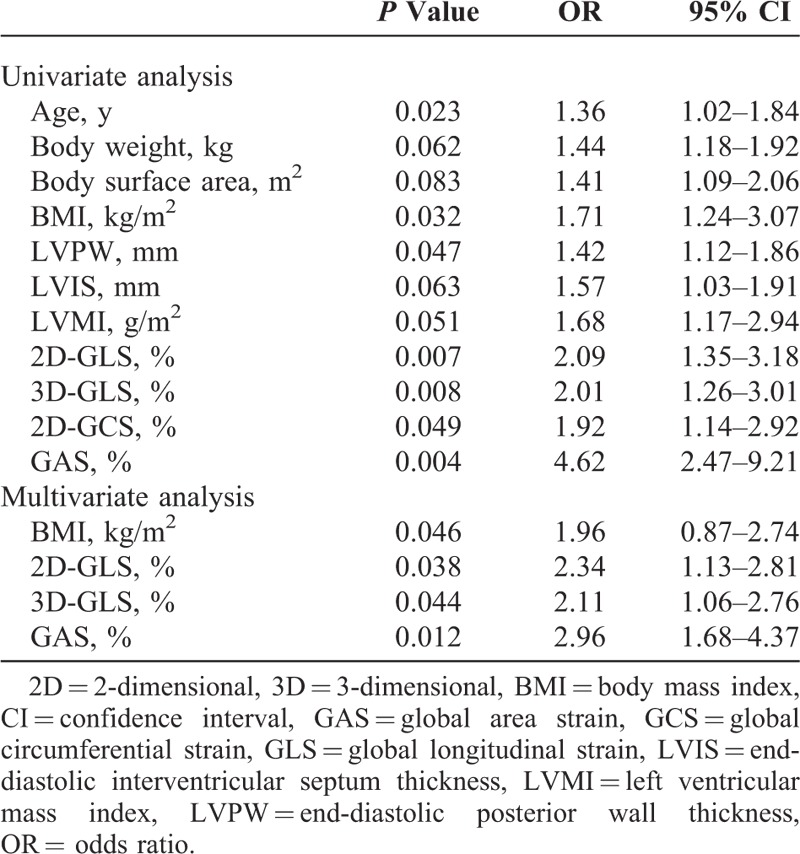
Univariate and Multivariate Analysis of Parameters Associated With Hypercholesterolemia

## DISCUSSION

The main findings of the present study were as follows: hypercholesterolemic children and adolescents have abnormal LV systolic and diastolic deformation parameters, obesity showed an additive adverse effect on LV strain parameters as well as impairment in RV indices in our young patients with lipid abnormalities, and both 2D and 3D speckle tracking techniques have advantages and disadvantages and could be used as methods of screening for LV abnormalities in this subpopulation. To the best of our knowledge, this is the first study to report the comparative use of 2DSTE and 3DSTE in normal-weight and obese hypercholesterolemic children and the additional burden of obesity on ventricular function.

### Previous Studies

Various authors demonstrated abnormal endothelial function and increased intima-media thickness in children with familial hypercholesterolemia,^14,15^ but they did not assess the effect of isolated hypercholesterolemia on cardiac morphology and function. Other authors using 2DSTE^8^ showed abnormal LV longitudinal and circumferential systolic deformation parameters in children with heterozygous familial hypercholesterolemia despite normal ejection fractions and excluded the possibility that these abnormalities could be related to systemic arterial hypertension. It has also been shown^9^ that LV 2D speckle tracking longitudinal strain was lower in children and adolescents with body mass index >95th percentile, even in the absence of other comorbidities, indicating that an adverse effect on LV function is an early finding in obesity.

### Cardiac Dysfunction in Hypercholesterolemia

Heart diseases such as coronary artery disease, hypertensive heart disease, diabetic cardiomyopathy, and hypercholesterolemic cardiomyopathy can directly or indirectly cause cardiac macrovascular and/or microvascular abnormalities. Atherosclerotic plaques located in the proximal and middle portions of the coronary arteries have been described in adult patients with heterozygous familial hypercholesterolemia.^4^ Experimental studies showed that hypercholesterolemia can lead to cardiac hypertrophy by several mechanisms,^16,17^ such as increased plasma concentration of endothelin-1, leading to vasomotor alterations, activation of the hypertrophic signaling pathways in cardiomyocytes, and increased cardiac oxidative stress. An association between hypercholesterolemia and downregulation of connexin-43 expression inducing vascular injury and myocardial contractile dysfunction has also been reported.^18^ It has also been shown that dietary hypercholesterolemia induces a “cholesterol cardiomyopathy”^19^ characterized by systolic and diastolic dysfunction presumably related to alterations in the membrane lipid bilayer and intracellular calcium handling and the contractile changes associated with cholesterol feeding are similar to those seen in models of myocardial hypertrophy but without the accompanying hypertrophy or hemodynamic overloading.

In the present study, hypercholesterolemic patients had increased LV mass index as well as mild systolic and diastolic abnormalities. No correlation was found between strain parameters and indexed LV mass. This is in agreement with previously reported data^8^ showing that reduced values of myocardial deformation properties are not a simple consequence of LV hypertrophy. If deformation abnormalities occur in the presence of LV hypertrophy, they can differ depending on the etiology of hypertrophy. In patients with hypertensive heart disease, a reduction of longitudinal deformation occurs with preserved circumferential deformation.^20^ In hypertrophic cardiomyopathy, myocardial deformation is usually impaired along 3 planes,^21^ whereas in athletes, myocardial strain may be either normal or increased in the presence of LV hypertrophy.^22^

A significant inverse correlation was found between longitudinal deformation and LDL cholesterol level, and a significant higher correlation was found between GAS obtained by 3DSTE and LDL cholesterol level. Several studies have validated 3DSTE, both in vitro and in vivo, against reference techniques such as sonomicrometry and magnetic resonance imaging tagging.^23,24^ Area strain is a combination of longitudinal and circumferential function and has already been validated as a useful measurement.^25,26^ Because it has integrated 2-directional components of LV myocardial deformation (longitudinal and circumferential), GAS might decrease the tracking error and emphasize synergistically the magnitude of deformation; thus, it is reasonable to expect that deteriorated LV function can be detected at an earlier stage by using 3D speckle tracking analysis in comparison with one-directional strain. Among the 4 measured strains (longitudinal, circumferential, radial, and area strains), we found that GAS had the best correlation with LDL cholesterol level and the highest discriminating power compared with normal controls, and this suggests a superiority of GAS over the conventional strain parameters in detecting early LV systolic dysfunction.

As in previous reports,^25,26^ we had a satisfactory correlation between 2DSTE and 3DSTE for GLS and GCS, but the correlation was poor for GRS. One reason may be that GRS values show a greater variability because they are calculated by both endocardial and epicardial speckle tracking data, whereas GLS and GCS are estimated only by endocardial tracking. Another reason might be the fact that the spatial motion gradient is calculated over a small region due to the limited wall thickness in combination with limited spatial resolution of the image.

Both 2DSTE and 3DSTE showed to be more sensitive to the detection of subtle myocardial damage compared with conventional indices of LV function.^27,28^ The superiority of 3DSTE over 2DSTE for the evaluation of all 3 components of LV deformation (GLS, GCS, and GRS) has been questioned.^29^ The 3D mode avoids foreshortening of apical views, consumes less time in data acquisition, helps to solve the problem of out-of-plane motion present in the 2D modality tracking motion of speckles in all 3 dimensions, and has good reproducibility as an automated method as shown by lower intraobserver and interobserver variabilities. However, this advantage is achieved at the expense of lower volume rate that might alter the correlations with measurements obtained by 2DSTE.

Our study is in keeping with previous reports showing that 3DSTE provides global and regional^23,26,30^ longitudinal and circumferential strain values that are comparable with the ones obtained from 2DSTE, even though they are not interchangeable with each other for various reasons. First, GLS was smaller on 3DE than 2DE imaging, and the lower longitudinal strain values may be explained by the twisting of the heart and out-of-plane rotation of myocardial segments on 2DE imaging.^30^ Second, the differences of strain values between the 2 methods were higher in the basal and apical segments than in the midventricular ones. These findings can be attributed to the diverging ultrasound beams toward the base that cause worse spatial resolution. In addition, these segments move at the highest velocities during the cardiac cycle, and this affects the accuracy of measurements due to the low frame rate of the current 3D echocardiography data sets. The problems of tracking in terms of apical segments can likely be attributed to the near-field artifacts or falling out of the field of view^30^ as it occurs in very lean subjects where the heart is close to the chest wall and further increasing the field of view is not possible unless decreasing frame rate which is unacceptable.

### Cardiac Dysfunction in Obesity

We also showed a correlation between deformation indexes and body mass index. Several mechanisms have been proposed to explain ventricular dysfunction in obesity, such as an increased mass in response to a larger intravascular volume, increased preload, and increased afterload.^31–33^ The mechanisms of cardiac remodeling with obesity are complex,^32^ and a major obstacle in attempts to characterize “obesity cardiomyopathy” is the prevalence of comorbid disorders such as insulin resistance, systemic hypertension, obstructive sleep apnea, type 2 diabetes mellitus, and physical inactivity. However, long-term follow-up studies found that obesity was associated with coronary artery disease independently of other cardiovascular risk factors,^34^ and other data suggested that overweight and obesity in young adults accelerate the progression of atherosclerosis before the appearance of clinical manifestations.^32,35^ The underlying pathophysiologic mechanisms that could lead to an increased risk for coronary artery disease include obesity-mediated free fatty acid turnover,^34^ obesity-mediated reduction in insulin sensitivity,^34^ induction of a hypercoagulable and hyperinflammatory state,^36^ and increased endothelial prostanoid-mediated vasoconstriction.^37^

Obesity showed an additive adverse effect on LV strain parameters in our young patients with lipid abnormalities, and this is reasonable on the basis of the above considerations. Previous studies in obese dyslipidemic children reported reduced systolic LV deformation characteristics, early vessel wall changes, and increased arterial stiffness suggesting an abnormal ventricular–vascular interaction.^34^ Subclinical changes in LV systolic and diastolic function have been described in obese adults^38^ and children.^9^ Children may show early cardiovascular dysfunction as a result of their excess adiposity, independently of other obesity-related comorbidities such as insulin resistance and dyslipidemia.^1^ Our data found LV systolic and diastolic abnormalities in obese dyslipidemic children. Systolic strain impairment was higher in the obese group compared with normal-weight hypercholesterolemic patients, indicating that this metabolic abnormality exerts an independent effect on LV function. Early diastolic strain rate is recognized as one of the markers of diastolic dysfunction and was lower in the hypercholesterolemic patients compared with controls, suggesting an incipient abnormal relaxation pattern. Because an abnormal E/E′_STE_ ratio is associated with elevated filling pressure, it is not surprising that this ratio remained within the normal range in normal-weight hypercholesterolemic children and adolescents and was higher in the obese group in whom increased mass and LV impairment were more severe.

An incipient RV dysfunction has also been shown in obese adults and children.^9,39,40^ In the pediatric population, the few papers that have analyzed RV in obesity showed different results. Potential reasons for this discrepancy may include the echocardiographic methods used, the differences in sample size, and the severity and duration of obesity. In the present study, we found a decreased RV free-wall strain and strain rate compared with controls, and these results are similar to those reported in nonhypertensive obese children using strain rate TDI.^39^ Other authors^9^ have described significantly higher RV strain and SR values in the obese patients, and it was speculated that, different from the LV function, systolic RV function was not an early abnormality in obesity and can be initially masked by the hypervolaemia state present in obesity because of an increase in preload, which would influence RV strain and strain rate.

### Clinical Implications

Our study shows the presence of LV deformation abnormalities in hypercholesterolemic children and adolescents and an accentuation of LV changes as well as an additional RV involvement in obese patients. We have also shown that 3DSTE allowed more rapid image acquisition and analysis because a single volumetric acquisition is required for this type of analysis, and this is convenient for any method to be applied clinically. Although 2D techniques require multiplane acquisitions for analysis, 3DSTE allows a reduction in acquisition time through the acquisition of the entire 3D volume data set from a single apical view over several cardiac cycles, and the postprocessing analysis performed allows derivation of all 4 components of 3D strain from a single analysis. Moreover, a further advantage of 3D-STE is the use of area strain for global LV function assessment, which is a novel index in addition to conventional strain parameters. However, 2D strain modalities are preferable whenever wall tracking tends to be suboptimal as well as in the assessment of RV function. Thus, our 2DSTE and 3DSTE data can be useful to critically assess not only the potentialities but also the actual limitations of speckle tracking echocardiography in the daily clinical practice. Overall, these findings provide additional evidence to induce physicians to manage hypercholesterolemia and obesity even at a young age and look for early detection of cardiac dysfunction in view of its potential reversibility.

### Limitations

Currently, 2 important technical limitations of 3DSTE are that the speckle tracking analysis is highly dependent on image quality, especially endocardial boundary delineation, and its low frame rate may lead to miscorrelation among frames and affect strain data accuracy. The low temporal resolution affects the ability to track anatomic details frame by frame and requires multibeat (6 beats) acquisitions. Although single-beat 3-dimensional STE data sets could have been acquired, image quality of single-beat acquisitions is not currently on an equal level with image quality of the multibeat acquisitions. Further research leading to improvements in both hardware and software is required to assess the feasibility of 3DSTE and the relative importance of current limitations such as low frame rates and suboptimal image quality.

The sources of variability are a further limitation, because the contribution to the overall coefficient of variation is not only related to the reader or the operator but also to beat-to-beat variability in single-beat and multibeat acquisitions. Consequently, efforts should be directed to utilize this information in developing more robust acquisition techniques and strategies.

Moreover, there is only limited experience cross-comparing intervendor differences in 3DSTE measurements.^41^ The use of a single vendor is appropriate for early research applications, but prevents widespread clinical applications in large populations across multiple imaging platforms and institutions.

Additionally, this was a relatively small observational study in a single-center protocol; thus, the prognostic implications of this mild LV dysfunction in order to improve the risk stratification of this subpopulation were not assessed and a larger study is required to confirm our findings.

### Conclusions

Dyslipidemia and obesity are associated with myocardial deformation changes as assessed by 2DSTE and 3DSTE in patients with no other cardiovascular risk factors or structural cardiac abnormalities. Obese dyslipidemic children and adolescents present greater impairment in LV strain parameters and impairment in RV strain compared with normal-weight dyslipidemic patients. Larger long-term studies are necessary to confirm the clinical importance of these results.
